# Anatomically based lower limb nerve model for electrical stimulation

**DOI:** 10.1186/1475-925X-6-48

**Published:** 2007-12-17

**Authors:** Juliana HK Kim, John B Davidson, Oliver Röhrle, Tanya K Soboleva, Andrew J Pullan

**Affiliations:** 1 Bioengineering Institute, The University of Auckland, Private Bag 92019, Auckland, New Zealand; 2 The Department of Engineering Science, Faculty of Engineering, The University of Auckland, Private Bag 92019, Auckland, New Zealand; 3 AgResearch Limited, Ruakura Research Centre, Private Bag 3123, Hamilton, New Zealand

## Abstract

**Background:**

Functional Electrical Stimulation (FES) is a technique that aims to rehabilitate or restore functionality of skeletal muscles using external electrical stimulation. Despite the success achieved within the field of FES, there are still a number of questions that remain unanswered. One way of providing input to the answers is through the use of computational models.

**Methods:**

This paper describes the development of an anatomically based computer model of the motor neurons in the lower limb of the human leg and shows how it can be used to simulate electrical signal propagation from the beginning of the sciatic nerve to a skeletal muscle. One-dimensional cubic Hermite finite elements were used to represent the major portions of the lower limb nerves. These elements were fit to data that had been digitised using images from the Visible Man project. Nerves smaller than approximately 1 mm could not be seen in the images, and thus a tree-branching algorithm was used to connect the ends of the fitted nerve model to the respective skeletal muscle. To simulate electrical propagation, a previously published mammalian nerve model was implemented and solved on the anatomically based nerve mesh using a finite difference method. The grid points for the finite difference method were derived from the fitted finite element mesh. By adjusting the tree-branching algorithm, it is possible to represent different levels of motor-unit recruitment.

**Results:**

To illustrate the process of a propagating nerve stimulus to a muscle in detail, the above method was applied to the nerve tree that connects to the human semitendinosus muscle. A conduction velocity of 89.8 m/s was obtained for a 15 *μm *diameter nerve fibre. This signal was successfully propagated down the motor neurons to a selected group of motor units in the muscle.

**Conclusion:**

An anatomically and physiologically based model of the posterior motor neurons in the human lower limb was developed. This model can be used to examine the effect of external stimulation on nerve and muscle activity, as may occur, for example, in the field of FES.

## Background

Functional Electrical Stimulation (FES) is a technique, using external electrical stimulation, which is capable of rehabilitating or restoring functionality of skeletal muscles that has been paralysed, for instance, by central nervous system lesions. The stimulation electrode can be located on the skin or implanted within the body to directly stimulate the muscles or nerves [1].

The direct stimulation of nerve trunks offers many advantages over transcutaneous or intramuscular stimulation. These advantages include the potential for controlling many muscles with a single implant, lower power requirements and the ability to place the electrode far from the contracting muscles [2]. Also, further development on nerve electrodes, like FINE (flat interface nerve electrodes) [2], allow selective stimulation of fascicles in peripheral nerves, which could be important in selectively activating individual muscles as well as reducing muscle fatigue that often occurs with FES.

Despite the success of FES achieved to date, there are a number of questions that cannot yet be completely answered. For instance (i) how and where should the electrodes be arranged on the nerves to achieve a specific muscle response? (ii) what stimulation protocol should be used on a nerve to achieve a desired level of force while minimising the symptoms of muscle fatigue?

The answers to these questions strongly depend on how well one can predict the effects of external stimuli with respect to the initiation and propagation of nerve impulses within a nerve tree. An effective way of analysing such nerve responses is by appealing to a computational nerve model which is based on anatomical features and the electrophysiological behaviour of neurons.

Early nerve models used the Hodgkin-Huxley equations which are derived from experimental data on squid giant axons [3]. A decade later, Fankenhaeuser and Huxley developed a new model, the so-called Fankenhaeuser-Huxley (or FH) model that was based on experimental data from myelinated nerve fibres of the toad Xenopus laevis [4]. In 1976 McNeal developed the first nerve model for a myelinated fibre using stimulus electrodes outside a fibre. In 1985, the SENN model (Spatially Extended Nonlinear Node model), based on amphibian data, was presented by Reilly et al. [5]. This model can accommodate arbitrary spatial and temporal distributions of the extracellular potentials and thus it makes it possible to simulate the behaviour of myelinated nerve fibres for different extracellular electrode configurations and stimulus protocols. In 1987, Sweeney et al. [6] published the first model for warm blooded nerves using the data of Chiu et al. [7], which stem from myelinated nerves of rabbits. This model has subsequently been called the CRRSS model, named after the investigators Chiu, Ritchie, Rogert, Stagg and Sweeney. Schwarz and Eikhof also presented their model, which was based on data from rat nerves [8]. In 1992, a modified SENN model was presented by Frijns et al. [9]. This model has adapted the SENN model to fit mammalian nerve fibre data by taking into account the influence of body temperature on nerve kinetics. In 2001 McIntyre et al. [10] described a model of mammalian nerve fibres using the concept of a double cable that had been earlier proposed by Halt and Clark [11].

While all the above mentioned models have previously been used within the field of FES [12-15], the CRRSS model is by far most popular one. This nerve model describes mammalian nerves more realisticly than earlier ones and it does not require the use of a double cable structure. For all these reasons this paper uses the CRRSS model in order to examine signal propagation in an anatomically based representation of the nerves in the human lower limb.

To the authors' knowledge, none of the previous works within the field of FES have used an anatomically based geometry of the nerve tree in the lower limb. The geometry of the nerve, however, plays a crucial role in analysing functional responses of muscles, e.g. the order of recruitment of muscles after an external source initiated a nerve stimulus. Therefore we have created an anatomically based geometric model of the posterior motor neurons in human lower limb to analyse the nerve responses more realistically and to allow us to look at the effect of external stimulation on nerve and muscle activity in the future.

## Methods

### Construction of the model of the lower limb nerves

#### Anatomy

The sciatic nerve is a major signal supplier to the legs and impulses conducted through its branches provide movement and feeling to the hip, thigh, knee, calf, ankle, foot and toes. The sciatic nerve starts from the lower spine and passes through the pelvic area and down through the hip and back of the leg branching into the tibial and common fibular nerves around the popliteal fissa area. The tibial nerve descends down to the feet along the back of the leg and the common fibular nerve is separated into the superficial and the deep fibular nerve descending along the front of the knee down to the feet [16,17]. Our model of the nerve tree starts from the sciatic nerve and forms nerve branches (tibial, common fibular, superficial fibular, deep fibular nerves) and the branches to muscles – hamstring (semitendinosus, (part of) adductor magnus, semimembranosus, short biceps, long biceps), calf muscles (gastrocnemius, soleus), tibialis posterior, flexor digitorum longus, flexor hallucis longus, fibular longus, fibular bravis, tibialis anterior, extenxor digitorum longus, and the extenxor hallucis longus.

#### Digitising and fitting of main nerve trunk

**Figure 1:**
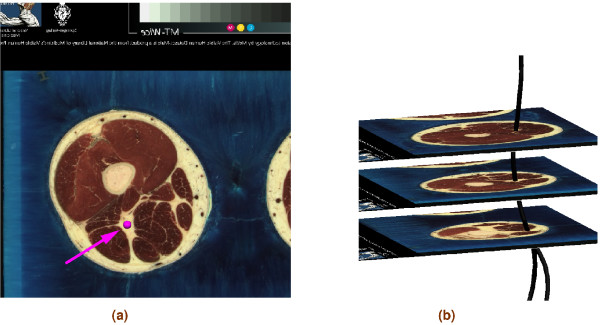
**Digitising and fitting**. An illustration of the nerve digitisation and fitting process. (a) Slice VM1195 from the Visible Man project with the sciatic nerve identified with a single point and arrow. (b) A fitted representation of part of sciatic nerve from the VM dataset and two of its branches- the tibial and common fibular nerves.

The main nerve trunk of the sciatic, tibial, common fibular, deep fibular and superficial fibular nerves was created manually by digitising the images of the Visible Man (VM) dataset, which has a spatial resolution of 0.33 mm per pixel and 1 mm distance between slices [18]. The digitisation was performed from slice VM0850 to VM1700. In cases where the main nerve trunk was not visible in the images, approximate locations of the nerves were identified using standard anatomical texts e.g. [19]. Furthermore, [20-22] were used to determine the location and the number of branches separating from the main nerve trunk to connect to a particular muscle. One-dimensional cubic Hermite finite elements were fitted to a point cloud, which was obtained by a manual digitisation process. The fitting process is described in [23,24]. The fitted nerve tree of the main trunk contains 145 elements. Figure 1 shows the VM slices used in the nerve digitisation process as well as part of the fitted model.

#### Nerve entry points

The biggest challenge in constructing a complete nerve tree from the images of the VM was the fact that nerves smaller than 1 mm cannot be identified. Hence an algorithm needed to be developed that connects the main nerve trunk with the motor endplates of a muscle.

Motor endplates are the flattened ends of motor neurons that transmit neural impulses to a muscle. While the motor endplates cannot be determined directly from the VM images, their locations can be approximated using anatomical features specific to human skeletal muscle. In humans, muscle fibres run in most human leg muscles from tendon to tendon and the endplates are situated around the midpoint of the muscle fibre (the exceptions are the satorious and the gracillis muscles). Moreover, the innervating endplates form either a transverse band through the middle of the muscle or a concave endplate band depending on the shape of the specific muscle [25]. This is also true for the semitendinosus muscle which is separated into two areas by an internal tendon. Thus the semitendinosus muscle has two bands of motor endplates and two separate nerve branches leading to the muscle. Using the geometrical models of the muscles in the lower limb the nerve entry points can hence be computed by determining the middle points of muscle fibres that run from tendon to tendon.

**Figure 2:**
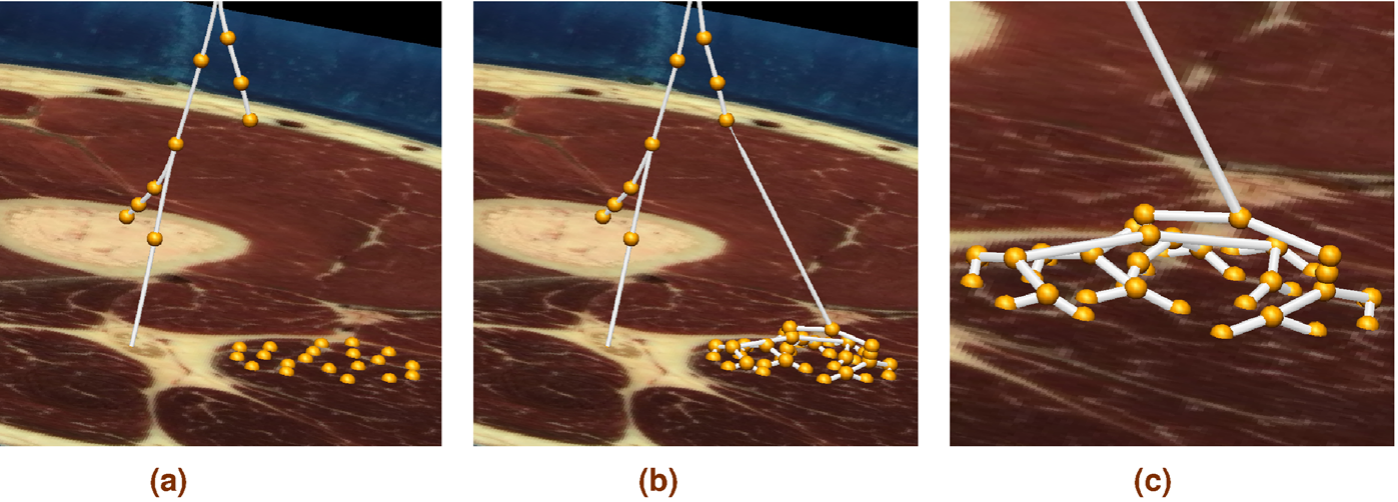
**Connection to nerve entry points**. Illustration of the process of connecting the fitted nerve trunk to the nerve entry points. (a) Nerve entry points were positioned at the appropriate locations on the VM images. (b) An automatic nerve tree was generated and connected to the main nerve trunk. (c) An enlarged picture of the generated nerve tree.

The knowledge of the nerve entry points allowed the construction of the whole nerve model by means of a tree growing algorithm. The tree was constructed by connection of two points to a single entity. This process is illustrated in Figure 2. Note that the grouping of nerve entry points should depend on its association with a particular motor unit, which is the basic functional unit of a skeletal muscle. The number of fibres in a motor unit varies greatly, and can range from 6 to 2000 depending on the types of control necessary [26].

**Figure 3:**
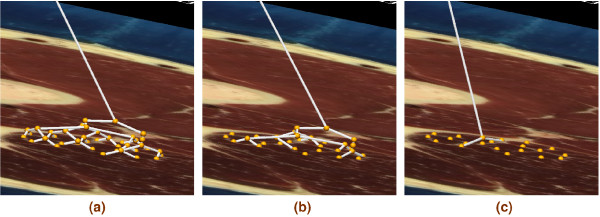
**Motor unit recruitment**. Generating nerve trees on the semimembranosus muscle from (a) 20 out of 20 motor entry points (100% recruitment). (b) 10 out of 20 motor entry points (50% recruitment) (c) 2 out of 20 motor entry points (10 % recruitment). A maximum of 20 motor units was used for illustrative purposes.

A detailed description of muscle motor units requires, for instance, a total of 580 motor units with 1720 fibres per motor unit for the gastrocnemius muscle or 445 motor units with 610 fibres per motor unit for the tibialis anterior muscle [26]. With this number of fibres and motor units a complete simulation of activation for the entire muscle is an extremely large computational job that is not currently feasible. However, individual skeletal muscle fibres are electrically isolated and the propagating action potential is confined to single fibres. It is not thus necessary to include a detailed muscle fibre and motor unit description within our model to demonstrate the feasibility of a whole nerve model. Therefore, a lower number (20–128) of motor units and one fibre per motor unit was used to generate the results shown below. This assumption, and the binary type nature of the tree constructed from nerve entry points, allow one to easily choose the number of active fibres within a muscle. We used a graded recruitment, which was consistent with the normal functioning of skeletal muscle. Here, we modelled the graded recruitment by selecting a certain percentage of motor unit points randomly out of the total motor units given in a muscle and connecting these points to the nerve tree. An illustration of this is given in Figure 3, which shows nerve trees for 100%, 50% and 10% recruitment of motor units in the semimembranosus muscle. For illustrative purposes only 20 motor units were depicted.

**Figure 4:**
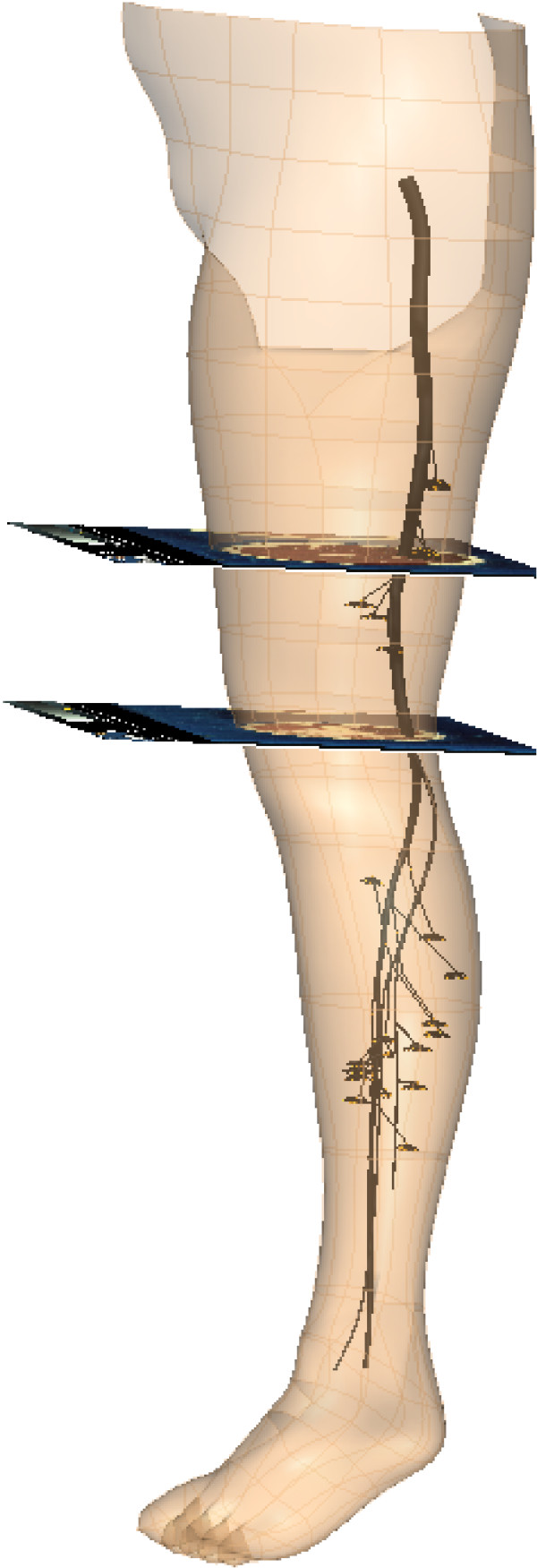
**Completed nerve tree**. Completed nerve tree in left lower leg, together with two of the images from the Visible Man dataset. The circled bits are the endplates for muscles.

Figure 4 shows the completed nerve model inside the left lower limb with two of the VH images used in the model construction.

### Signal propagation

Each fibre in a nerve is capable of generating an action potential which can be propagated along its length without loss of amplitude, duration, or velocity. The individual fibres respond to stimulation according to the all-or-none principle: If the neuron does not reach the critical threshold level, then no action potential will be generated. On the other hand, if the threshold level is reached, an action potential of a fixed size occurs.

**Figure 5:**
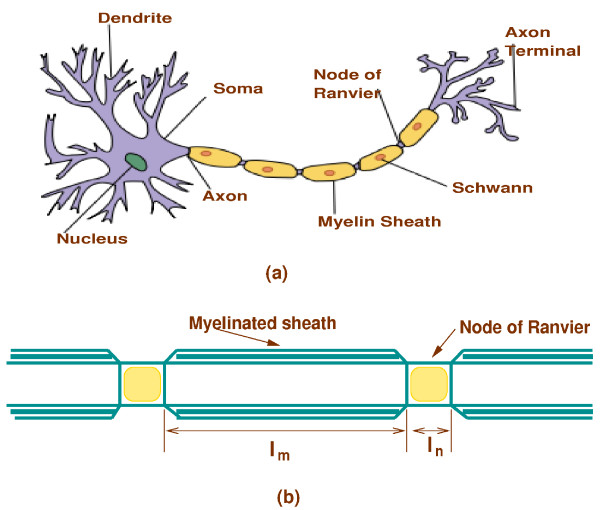
**Myelinated nerve and cable model**. (a) A myelinated nerve (b) A simplified representation of the axon of a myelinated nerve by considering the axon as a cylinder with nodes of Ranvier at regular intervals along the cylinder and with myelinated sheaths between the nodes of Ranvier. The parameter *l*_*m *_represents the length of the myelinated sheath and *l*_*n *_represents the length of the node of Ranvier.

For myelinated nerve fibres, axons are encased by Schwann cells which wrap a myelin sheath around the surface of the axon and have nodes of Ranvier which are regularly spaced along the axon. This is depicted in Figure 5(a) (reproduced from [27] with permission under GFDL). Since myelin sheaths provide insulation to the nerve, within the myelinated part of the nerve, action potentials can only be elicited at the nodes of Ranvier. Figure 5(b) shows a simplified representation of the axon of a myelinated nerve by considering the axon as a cylinder with nodes of Ranvier at regular intervals along the cylinder and with myelinated sheaths between the nodes of Ranvier.

As an axon reaches the vicinity of the skeletal muscle fibre it loses its myelin sheath [28]. The non-myelination and the small diameter of the nerve endings do both contribute to the reduction of the conduction velocity by a factor of between 1/50 and 1/100 when compared to the speed at which the impulse travels in the myelinated unbranched parent axon [29]. In our model, however, the details of the nerve terminals were not modelled as the main focus of this study is on the conduction velocity of the unbranched axon. Hence, we assumed that the nerve fibre was myelinated along its entire length and the diameter of the fibre was constant along the whole nerve tree.

In the CRRSS model, the nodes of Ranvier are modeled by voltage-dependent sodium channels, voltage-independent leakage channels and nodal capacitance. There are no voltage-dependent potassium channels in the ionic current equations of the CRRSS model since these channel types are scarce at nodes of Ranvier in mammalian myelinated nerve fibres. Hence action potential repolarization in mammalian myelinated axons is attributed to rapid sodium inactivation and a large leakage current [7]. A complete list of the CRRSS ionic currents equations together with the corresponding list of constants and variable names is given in APPENDIX A (See Additional file 1: appendixa).

With our proposed focus on external (extracellular) stimulation, the bidomain model was chosen to represent the electrical activity occurring along the nerve tree. The bidomain equations have the advantage that they explicitly model the extracellular potential and are given by

∇·((*σ*_*i *_+ *σ*_*e*_) ∇ *φ*_*e*_) = -∇·(*σ*_*i *_∇ *V*_*m*_) + *I*_*stim*_,

∇⋅(σi∇Vm)+∇⋅(σi∇ϕe)=Am(Cm∂Vm∂t+Iion),
 MathType@MTEF@5@5@+=feaagaart1ev2aaatCvAUfKttLearuWrP9MDH5MBPbIqV92AaeXatLxBI9gBaebbnrfifHhDYfgasaacPC6xNi=xI8qiVKYPFjYdHaVhbbf9v8qqaqFr0xc9vqFj0dXdbba91qpepeI8k8fiI+fsY=rqGqVepae9pg0db9vqaiVgFr0xfr=xfr=xc9adbaqaaeGacaGaaiaabeqaaeqabiWaaaGcbaGaey4bIeTaeyyXICTaeiikaGccciGae83Wdm3aaSbaaSqaaiabdMgaPbqabaGccqGHhis0cqWGwbGvdaWgaaWcbaGaemyBa0gabeaakiabcMcaPiabgUcaRiabgEGirlabgwSixlabcIcaOiab=n8aZnaaBaaaleaacqWGPbqAaeqaaOGaey4bIeTae8x1dy2aaSbaaSqaaiabdwgaLbqabaGccqGGPaqkcqGH9aqpcqWGbbqqdaWgaaWcbaGaemyBa0gabeaakiabcIcaOiabdoeadnaaBaaaleaacqWGTbqBaeqaaKqbaoaalaaabaGaeyOaIyRaemOvay1aaSbaaeaacqWGTbqBaeqaaaqaaiabgkGi2kabdsha0baakiabgUcaRiabdMeajnaaBaaaleaacqWGPbqAcqWGVbWBcqWGUbGBaeqaaOGaeiykaKIaeiilaWcaaa@5EA9@

where

*σ*_*i *_: intracellular conductance

*σ*_*e *_: extracellular conductance

*φ*_*e *_: extracellular potential

*V*_*m *_: transmembrane potential

*I*_*ion *_: total ionic current, as given by the CRRSS model

*C*_*m *_: membrane capacitance

*A*_*m *_: the ratio of membrane surface area to the volume

*I*_*stim *_:extracelluar injected current

To use the governing two bidomain equations to model myelinated fibres, the left hand side of the equation (2) needed to be multiplied by *l*_*m*_*/l*_*n *_[30]. Here, *l*_*m *_is a internodal length (myelinated part) and *l*_*n *_is a nodal gap length (node of Ranvier)(Figure 5(b)).

The modified bidomain equations were discretised using the same finite different scheme as proposed in [30]. The resulting equations were then solved using an implicit numerical integration scheme (LSODA) [31,32] with a uniform spatial discretisation and a fixed time step of 0.001 *ms *as implemented within CMISS [33]. Solving the modified bidomain equations with values as given in [6] resulted in a slow conduction velocity. Therefore, the value of the axoplasm resistivity, the ratio of fibre diameter to axon diameter and the ratio of internodal length to axon diameter were changed in such a way that an appropriate action potential conduction velocity for humans was generated. In Waxman's review [34] of the conduction velocity in myelinated nerve fibres, he determined theoretically that to achieve maximal conduction velocity per axon diameter, the ratio of fibre diameter to axon diameter should be 0.6–0.7 and the ratio of the internodal length to axon diameter was found to be 100–200. These ratios change for different nerves and species.

Guided by the work in [35], we set the ratio of axon diameter to fibre diameter to be 0.67 and the ratio of internodal length to axon diameter to be 200.

#### Signal propagation to a muscle fibre

To illustrate the process of a propagating nerve stimulus to a muscle in detail, the above method was applied to the nerve tree that connected to the human semitendinosus muscle. The bidomain model was also used to simulate electrical propagation within the muscle fibres. Here though, the CRRSS model was replaced by the muscle cell model [36].

The muscle model [36] is a mathematical model of a single skeletal muscle fibre that is based on electro-physiological processes. This model describes fast and slow twitch fibre types and was developed to investigate and model the muscle fatigue. The parameters that were used in our paper and deviated from [36] are listed in Table 1.

**Table 1:** 

parameter	nerve	muscle	description
*C*_*m*_	2.5	1	capacitance/unit area(*μF/cm*^2^)
*R*_*i*_(1/*σ*_*i*_)	0.24	2.5	intracellular resistivity(*Ohmm*)
*R*_*e*_(1/*σ*_*e*_)	0.24	1.11	extracellular resistivity(*Ohmm*)
*A*_*m*_	444	20	The ratio of membrane surface to volume(1*/mm*)

## Results

**Figure 6:**
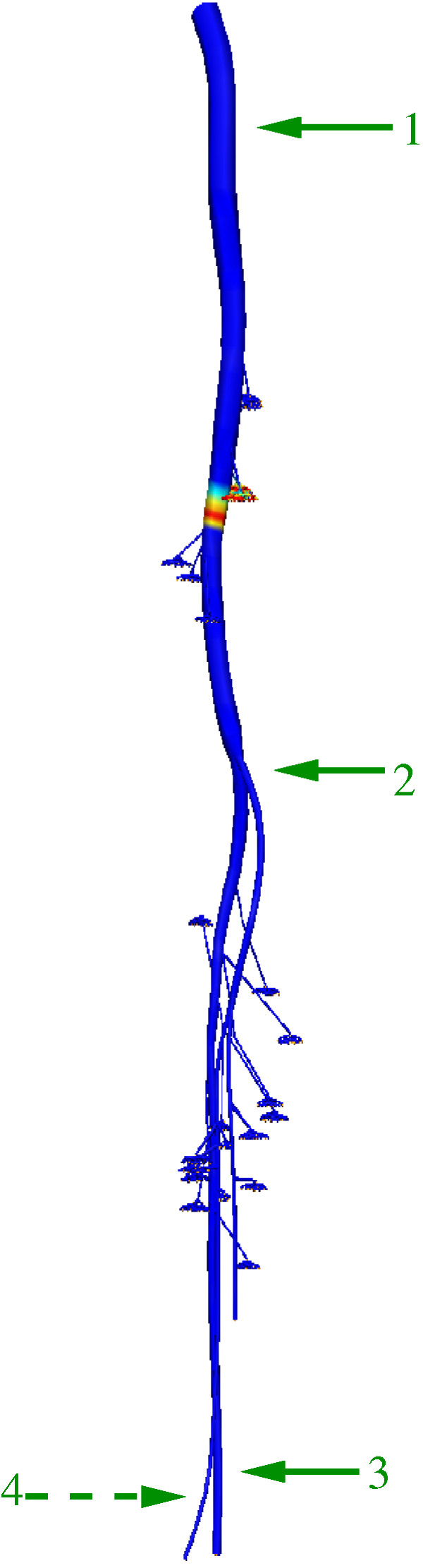
**Action potential propagation**. The entire nerve tree model. The coloured field between positions 1 and 2 represent an action potential that has been generated from an extracellular stimulus at the top of the tree. Action potentials at positions labelled 1 through 4 are given in Figure 6.

**Figure 7:**
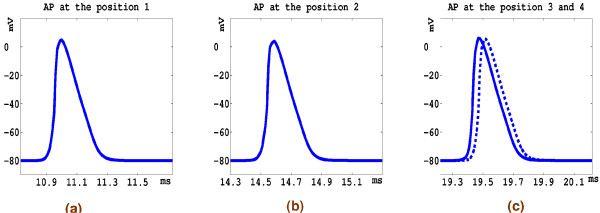
**Action potentials**. Action potential (AP) along the nerve tree. Positions are shown in Figure 5(a) AP at position 1. (b) AP at position 2. (c) AP at position 3 and 4.

We initially present results for the action potential propagation down the nerve tree. An initial cathodic extracellular rectangular current stimulus of 0.014 *μA *was imposed at a node at the beginning of the sciatic nerve for a duration of 0.2 ms. The value of the current stimulus was obtained by injecting a 4.0 × 10^4 ^*μA/cm*^3 ^source over a 3.5 × 10^-7 ^*cm*^3 ^volume. Note that there is no distance between the excitation monopolar electrode and the stimulated node. At this point in the nerve tree, the fibre diameter was set to be 15 *μm *based on [9], with a corresponding axon diameter of 10 *μm *and internodal length of 2.0 *mm *(since ratios of 0.67 and 200 had been set as discussed above). The axoplasm resistivity was set to be 0.24 *Ohmm*. Figures 6 and 7 depict the action potential propagation along the nerve tree. It can be seen that the computed action potentials at different positions along the nerves have the same shape, magnitude and duration. In our results, the magnitude of an action potential is approximately 85 mV and its duration is 0.32 *ms *with a rise time 0.07 *ms*. This duration compares favourably with the known duration of an action potential at 37°*C *in the single myelinated rat nerve fibre (approximately 0.3 *ms *[37]). Here, the calculation of the rise and fall times of the action potentials was done according to the method of [9].

The conduction velocity in the main nerve trunk was calculated by running the simulation for 10 ms and measuring the distance which the stimulus has propagated from a point close to the beginning of the sciatic nerve. The calculated conduction velocity was 89.8 *m/s*. This result agrees well with previously published theoretical results of 90 *m/s *[10,38]. It also agrees well with the experimental work of Boyd and Kalu [39], in which the relationship between conduction velocity and nerve fibre diameter was investigated experimentally using ten different muscle nerves in two cats.

In our work a conduction velocity of 85.35 *m/s *is predicted from their approximate relationship.

**Figure 8:**
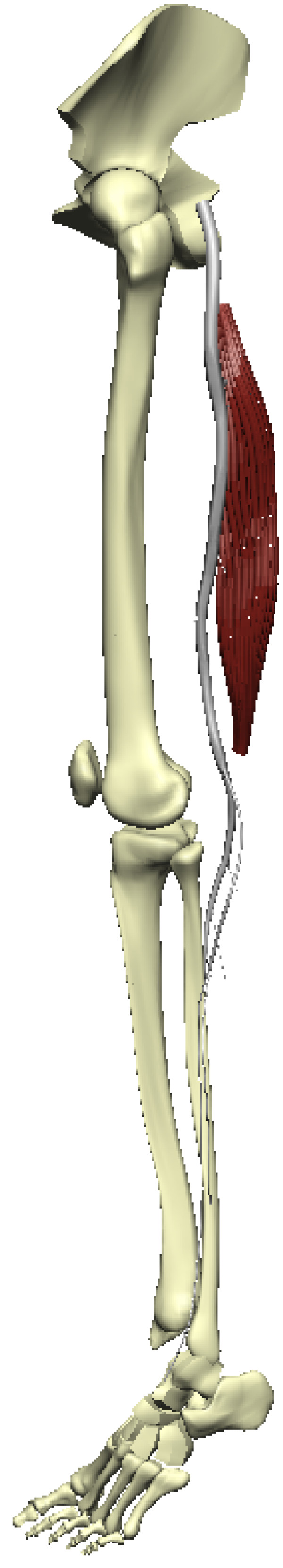
**Semitendinosus muscle fibres connected to nerve tree**. Semitendinosus muscle fibres connected to the two branches of the nerve tree.

**Figure 9:**
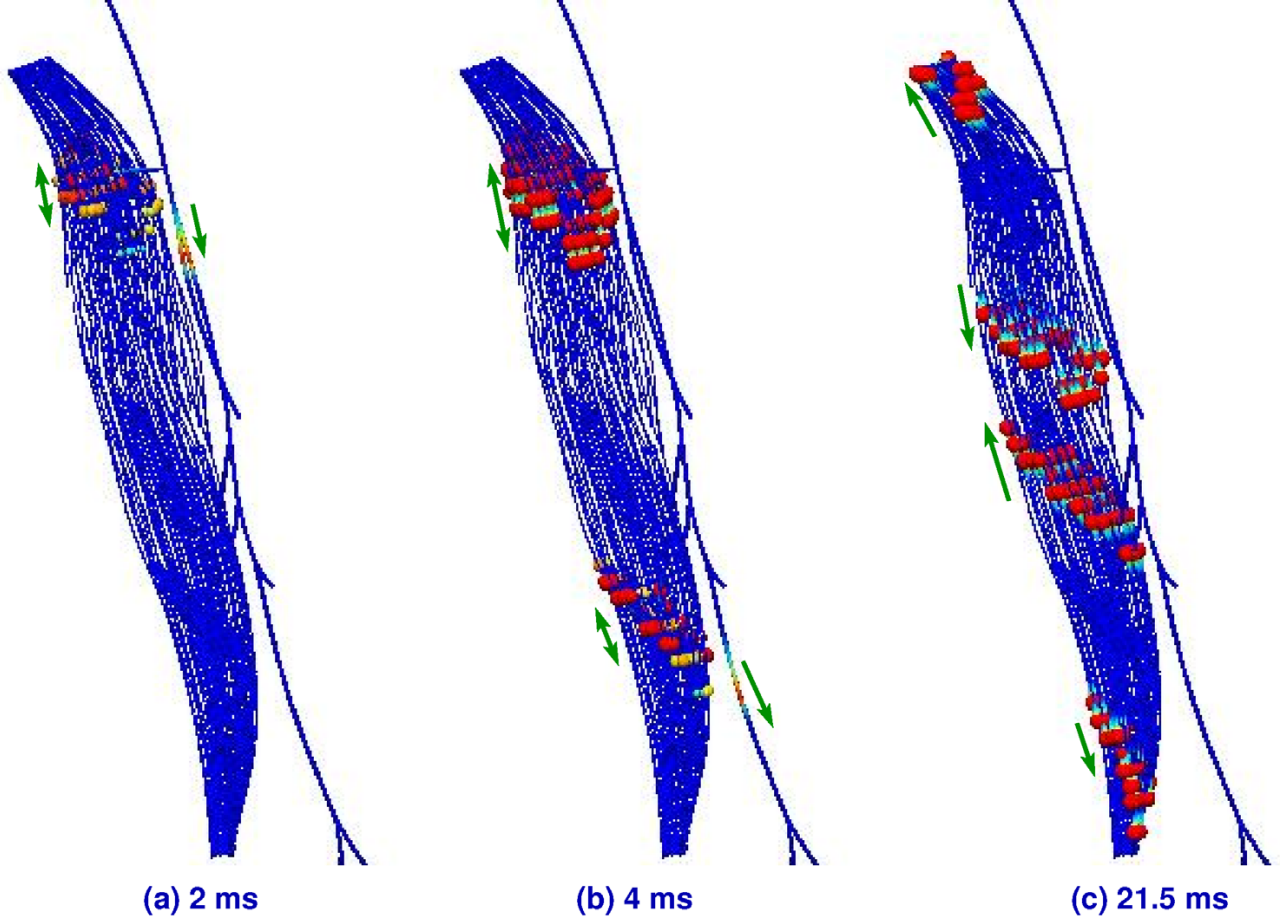
**AP propagation to the semitendinosus muscle**. Action potential (AP) along the nerve tree activating several motor units of the semitendinosus muscle. The timing reference given in each picture is the time since the stimulation at the beginning of the sciatic nerve and the direction of action potential propagation in both the nerve and the muscle is indicated by corresponding arrows. (a) AP has reached the proximal endplate of the muscle and begins depolarising fibres. (b) AP has reached the distal endplate of the muscle and continues down the nerve. APs continue to propagate along fibres at the proximal endplate. (c) AP depolarisation of muscle fibres spreads from both endplates.

To further validate our model with respect to the calculated conduction velocities, two further simulations, involving the parameters in Table 2, were considered. We chose 10 *μm *and 20 *μm *diameter fibres since the relationship between conduction velocities and fibre diameters investigated in [39] are between these two values. Axon diameters and internodal lengths are also chosen by the ratios we used for the 15 *μm *diameter fibre. For these parameters, our simulations predicted conduction velocities 120 *m/s *and 54 *m/s *for the 20 *μm *and 10 *μm *diameter fibres respectively. In [38], the approximate conduction velocities are predicted to be 120 *m/s *for a 20 *μm *diameter fibre and 57 *m/s *for a 10 *μm *diameter fibre, where as [39] predicts conduction velocities of 113.8 *m/s *for a 20 *μm *diameter fibre and 56.9 *m/s *for a 10 *μm *diameter fibre. In addition to the propagation of a nerve stimulus, we also simulated the resultant activity within the respective muscle. Figure 8 shows the semitendinosus muscle connected to the nerve tree. The signal propagation is illustrated in Figure 9, where an action potential was propagated along the nerve to initiate a contractile response within 128 muscle fibres embedded in the semitendinosus muscle. The semitendinosus muscle has an inscription in the middle of the muscle and thus two branches of the nerve tree were connected to the upper and the lower part of the muscle. In Figure 9, the nerve model was stimulated at the beginning of the sciatic nerve and an action potential propagated along the nerve trunk, its branches, through the motor endplates, and along the muscle fibres of the semitendinosus muscle.

**Table 2:** Simulation parameters for the 10 and 20 *μm *diameter fibres.

fibre diameter (*μm*)	axon diameter (*μm*)	internodal length (mm)	axoplasm resistivity (*Ohmm*)	*Am *(1*/mm*)
20	13	2.6	0.19	308
10	7	1.4	0.30	571

## Discussion

We have created an anatomically based geometric model of the posterior motor neurons of the human lower limb. The geometry was mainly based on data from the Visible Man dataset and was supplemented, where necessary, using existing anatomical and physiological texts. Using this geometrical representation, action potentials were simulated using a modified representation of the bidomain equations that incorporated the CRRSS ionic conductance model. When an appropriate external stimulus was applied, an action potential was propagated along the nerve tree with a constant velocity of 89.8 *m/s *for a 15 *μm *diameter fibre. Most importantly the shape of an action potential was not altered by the simulation. The computed action potential conduction velocity agrees well with previously published theoretical and experimental estimates [10,38,39].

The axoplasm resistivity we used for the 15 *μm *diameter fibre was lower than that used in the original CRRSS model (0.24 *Ohmm *compared to 0.547 *Ohmm *[6]). However, in the original CRRSS paper, an intracellular stimulus was used with a 10 *μm *diameter fibre. It has also been noted that resistivity values are difficult to determine experimentally and agreed values are not yet well established [40].

The amplitude of the computed action potential (85 *mV*) was lower that reported in [37] which is about 105 – 110 *mV*. However, it should be noted that the amplitudes from [37] are obtained using experimental data from rat and cat nerves as a guide. One of the reasons for the different amplitude between the published values and the one presented in this paper could be the sodium (Na) transient since the membrane potential of the neuron is greatly affected by Na rushing into the cell when voltage gated channels open. If there is less Na available outside the cell, the inward driving force is decreased, creating a smaller depolarisation. To examine this in more detail, we compared the Na Nernst potential *E*_*Na *_between our model and the model of Schwarz et al. [37]. In our case the value was 35.64 *mV *(which is the same as that used in the original CRRSS model) whereas the model of Schawrz et al. [37] used a value of 60 *mV*. Changing the Na Nernst potential to 55.64 *mV *in our model resulted in the action potential amplitude reaching approximately 100 *mV *together with an increased conduction velocity of 120 *m/s*. An additional increase of the axoplasm resistivity to 0.32 *Ohmm *decreased the conduction velocity to 90 *m/s *without any alteration to the action potential amplitude. Thus, the proposed model appears to be able to generate appropriate conduction velocities and action potential amplitudes using the axoplasm resistivity and Na Nernst potential. We note that currently good values for these parameters for the human motor nerves are not available.

One of the limitations of our model is the graded recruitment which was only mimicked by selecting a certain percentage of motor units and generating a nerve tree based on the motor entry points within these motor units. In other words, a graded recruitment was not achieved by the different strength of stimuli but by specifying the number of motor units to recruit a priori. This limitation can be overcome by modelling the sciatic nerve as a bundle of nerve fibres. With such a representation, each nerve fibre would be only connected to a specified set of motor units and the activation of each nerve fibre could then be defined according to the strength of stimuli. With our single nerve model replaced by a nerve bundle we can then investigate the relationship between surface electrode placement, stimulation strength, duration and activation of nerve fibres within the fibre bundle. The question of what external electrode configuration is required to achieve activation of a particular group of nerve fibres can then begin to be addressed. We intend to investigate this approach soon.

We have further shown that the nerve model can be connected to a skeletal muscle fibre model using the semitendinosus muscle as an illustration. The (neuron) action potentials propagated along the nerve to initiate a contractile response within the muscle. A novel approach of effectively bridging the scales of the electrophysiological cellular behaviour and the mechanical response of a skeletal muscle has been recently developed [41]. This combination can be used as a powerful tool to investigate and answer open questions in the field of FES, e.g the use of specific stimulation protocols to achieve a desired movement or to delay the fatigue process.

## Conclusion

This paper introduces an anatomically based geometric model of the posterior motor neurons in human lower limb based on data from the Visible Man dataset. Using this geometrical representation, action potentials were computed using a bidomain representation that incorporated the published ionic conductance model. The results indicate that this model can be used to look at the effect of external stimulation on nerve and muscle activity in the field of FES.

## Competing interests

The author(s) declare that they have no competing interests.

## Authors' contributions

Author JHKK created the human lower limb nerve model based on VH images, performed the various simulations required, analyzed the results and drafted the manuscript. Author JBD created the muscle fibres and connected it to the nerve model and performed simulations required. Author OR helped to create the semitendinosus muscle and revised the manuscript. Author TKS contributed to the muscle model development. Author AJP was actively involved in the supervision and development of the research and revised the manuscript. All authors read and approved the final version of the manuscript.

## Supplementary Material

Additional file 1 APPENDIX A. The equations, variables and constants used in our model are presented. Click here for file
