# Supplementary material for: Anatomically based lower limb nerve model for electrical stimulation
Source: Biomed Eng Online. 2007 Dec 17;6:48. doi: 10.1186/1475-925X-6-48 (PMC2228306; doi:10.1186/1475-925X-6-48)
Supplement: Additional file 1 — APPENDIX A. The equations, variables and constants used in our model are presented. [file 1475-925X-6-48-S1.pdf]

APPENDIX A

Equations

The ionic current equations of the CRRSS model can be written as

1. $I_{ion} = I_{Na} + I_L$

2. $I_{Na} = g_{Na} m^2 h (V_m - E_{Na})$

   $V = V_m - E_r$

   $\frac{dm}{dt} = \alpha_m (1 - m) - \beta_m m$

   $\alpha_m = \frac{97 + 0.363V}{(1 + exp(\frac{31 - V}{5.3}))}$

   $\beta_m = \frac{\alpha_m}{exp(\frac{V - 23.8}{4.17})}$

   $\frac{dh}{dt} = \alpha_h (1 - h) - \beta_h h$

   $\alpha_h = \frac{\beta_h}{exp(\frac{V - 5.5}{5})}$

   $\beta_h = \frac{15.6}{exp(\frac{24 - V}{10})}$

3. $I_L = g_L (V_m - E_L)$

Variables

I_{Na}	sodium current density
I_L	leakage current density
m	sodium channel activation
h	sodium channel inactivation

Constants

E_r	-80	resting membrane potential(mV)
g_{Na}	1445	maximum sodium channel conductance/unit area(mS/cm^2)
E_{Na}	35.64	Nernst potential for sodium channels(mV)
g_L	128	maximum leakage channel conductance/unit area(mS/cm^2)
E_L	-80.01	Nernst potential for leakage channels(mV)
l_n	1.5	nodal gap length(μm)
